# Improved analytical performance of plasma amyloid β assay using immunoprecipitation‐mass spectrometry

**DOI:** 10.1002/alz70856_097456

**Published:** 2025-12-24

**Authors:** Akihito Korenaga, Rie Yamamoto, Mayu Yoneyama, Hiroki Naito, Kazushi Ohta, Naoki Kaneko, Sadanori Sekiya, Shinichi Iwamoto, Koichi Tanaka

**Affiliations:** ^1^ Shimadzu Corporation, Kyoto, Japan; ^2^ Shimadzu General Services, Inc., Kyoto, Japan

## Abstract

**Background:**

The analysis of plasma biomarkers for Alzheimer's disease is non‐invasive and suitable for large‐scale screening. We have reported that the combination of three amyloid peptides in human plasma (Aβ1‐40, Aβ1‐42, APP669‐711) shows a good correlation with amyloid accumulation in human brain. (Nakamura et al., Nature 2018) Here, we report the results of the analytical validation of our plasma amyloid‐β assay using immunoprecipitation‐matrix‐assisted laser desorption/ionization time‐of‐flight mass spectrometry (IP‐MALDI‐MS) with a benchtop MALDI‐TOF mass spectrometer, instead of a conventional floor‐standing model previously reported (Yoda et al., AAIC 2021).

**Method:**

Using magnetic beads covalently bound with anti‐amyloid β antibody (clone 6E10), immunoprecipitation was performed on commercial plasma and/or synthetic peptide mixtures. The samples obtained from immunoprecipitation were measured by the benchtop MALDI‐TOF MS (Shimadzu/Kratos, UK). The peak intensities from amyloid‐related peptides were normalized using stable isotope‐labeled (SIL) Aβ1‐38 as internal standards. The biomarkers Aβ1‐40/Aβ1‐42 and APP669‐711/Aβ1‐42 were evaluated for intra‐assay and inter‐assay reproducibility. For dynamic range, spike recovery, and dilution linearity, the normalized intensities of each peptide were assessed.

**Result:**

Each biomarker values showed a coefficient of variation (CV) of less than 5% for both intra‐assay and inter‐assay reproducibility, which was better than the previous data with the CV of < 5% in some cases. Trueness of 100±20% was confirmed over the range from 15 to 150 pM for Aβ1‐40, and from 1.5 to 15 pM for Aβ1‐42 and APP669‐711, which was superior to the previous results showing the trueness of > 130% at the lowest concentration. In the spike recovery test, recovery rates ranged from 87% to 113% across two concentration levels (low and high). In the dilution linearity test, linearity ranged from 88% to 111% for 2‐fold and 4‐fold dilutions.

**Conclusion:**

These results indicate that immunoprecipitation‐mass spectrometry system using the benchtop instrument possesses sufficient stability and reliability for the measurement of amyloid‐β peptides in plasma, with improvements in reproducibility and trueness.

